# Protein kinase A activation by the anti-cancer drugs ABT-737 and thymoquinone is caspase-3-dependent and correlates with platelet inhibition and apoptosis

**DOI:** 10.1038/cddis.2017.290

**Published:** 2017-06-29

**Authors:** Natalia Rukoyatkina, Elke Butt, Hariharan Subramanian, Viacheslav O Nikolaev, Igor Mindukshev, Ulrich Walter, Stepan Gambaryan, Peter M Benz

**Affiliations:** 1 Sechenov Institute of Evolutionary Physiology and Biochemistry, Russian Academy of Sciences, St. Petersburg, Russia; 2 Institute of Experimental Biomedicine II, University Hospital Wuerzburg, Wuerzburg, Germany; 3 Institute of Experimental Cardiovascular Research, University Medical Center Hamburg-Eppendorf, Hamburg, Germany; 4 DZHK (German Centre for Cardiovascular Research) Partner Site Hamburg/Kiel/Lubeck, Hamburg, Germany; 5 Center for Thrombosis and Hemostasis (CTH), University Medical Center Mainz, Mainz, Germany; 6 DZHK (German Centre for Cardiovascular Research) Partner Site Rhine-Main, Mainz, Germany; 7 Department of Cytology and Histology, St. Petersburg State University, St. Petersburg, Russia; 8 Institute for Vascular Signalling, Centre for Molecular Medicine, Johann Wolfgang Goethe University, Frankfurt am Main, Germany; 9 DZHK (German Centre for Cardiovascular Research) Partner Site Rhine-Main, Frankfurt am Main, Germany

## Abstract

Chemotherapy-induced thrombocytopenia is a common bleeding risk in cancer patients and limits chemotherapy dose and frequency. Recent data from mouse and human platelets revealed that activation of protein kinase A/G (PKA/PKG) not only inhibited thrombin/convulxin-induced platelet activation but also prevented the platelet pro-coagulant state. Here we investigated whether or not PKA/PKG activation could attenuate caspase-dependent apoptosis induced by the anti-cancer drugs ABT-737 (the precursor of navitoclax) and thymoquinone (TQ), thereby potentially limiting chemotherapy-induced thrombocytopenia. This is particularly relevant as activation of cyclic nucleotide signalling in combination chemotherapy is an emerging strategy in cancer treatment. However, PKA/PKG-activation, as monitored by phosphorylation of Vasodilator-stimulated phosphoprotein (VASP), did not block caspase-3-dependent platelet apoptosis induced by the compounds. In contrast, both substances induced PKA activation themselves and PKA activation correlated with platelet inhibition and apoptosis. Surprisingly, ABT-737- and TQ-induced VASP-phosphorylation was independent of cAMP levels and neither cyclases nor phosphatases were affected by the drugs. In contrast, however, ABT-737- and TQ-induced PKA activation was blocked by caspase-3 inhibitors. In conclusion, we show that ABT-737 and TQ activate PKA in a caspase-3-dependent manner, which correlates with platelet inhibition and apoptosis and therefore potentially contributes to the bleeding risk in chemotherapy patients.

Chemotherapy-induced thrombocytopenia is a frequent problem in cancer patients. Besides the bleeding risk, thrombocytopenia limits chemotherapy dose and frequency. Well-known anti-cancer drugs such as oxaliplatin, or navitoclax and others induce thrombocytopenia,^1, 2^ at least in part by induction of apoptosis. In nucleated cells, apoptosis is characterized by the loss of mitochondrial membrane potential (ΔΨ_m_), the release of cytochrome C into cytosol, and subsequent caspase 9 activation. Caspase 9 then activates the effector caspases, 3 and 7.^3, 4^ The release of cytochrome C is tightly regulated by the B-cell lymphoma 2 (BCL2) protein family which consists of pro- and anti-apoptotic members, which promote or block the release of cytochrome C from mitochondria. These events are reliable hallmarks of cell damage observed during apoptosis.

Circulating platelets contain many components of the apoptotic machinery.^5^ Inhibition of anti-apoptotic BCL2 and B-cell lymphoma-extra large (BCL-X_L_) prevents platelet activation.^6^ Apoptotic and pro-coagulant, or highly activated, platelets display common characteristics, such as loss of mitochondrial membrane potential, microparticle formation, and phosphatidylserine (PS) exposure.^6^ However, the molecular mechanisms responsible for PS surface exposure in apoptotic and pro-coagulant platelets are different.^7, 8, 9, 10^ In apoptotic cells and platelets, surface PS exposure is triggered by caspase-dependent activation of the Xk-related protein family member (Xkr8).^10, 11^ In pro-coagulant platelets, activated with a combination of thrombin and collagen or calcium ionophore under low calcium conditions, PS surface exposure is triggered mainly by activation of calcium-dependent scramblase transmembrane protein 16F (TMEM16F).^11, 12^ Recently, we showed that pro-coagulant activity induced by strong platelet stimulation using a combination of thrombin/convulxin (Thr/Cvx) is inhibited by protein kinase A (PKA) and protein kinase G (PKG) activation.^13^ However, whether or not PKA/PKG activation can also inhibit platelet apoptosis induced by caspase-dependent apoptotic stimuli is not known.

Cyclic AMP (cAMP) and cyclic GMP (cGMP), acting via their target kinases, PKA and PKG, are major players in platelet inhibition. PKA and PKG phosphorylate several key substrates^14, 15^ and inhibit all agonist-induced platelet activation pathways including calcium release, integrin activation, granule release, shape change, adhesion, and aggregation.^16, 17^ In nucleated cells, both cAMP and cGMP can induce pro- and anti-apoptotic effects.^18, 19, 20, 21^

In our study, we used two anti-cancer substances, ABT-737 and thymoquinone (TQ) with different mechanisms of inducing apoptosis and compared them with Thr/Cvx triggered apoptotic-like events in platelets. ABT-737, a precursor of the oral derivate ABT-263 (navitoclax), is a potent mimetic of Bcl-2 homology 3 domain (BH3)-only proteins (including the Bcl-2 interacting mediator of cell death (Bim), BH3 interacting domain death agonist (Bid) and other proteins which are important in binding and neutralizing anti-apoptotic Bcl-2 family proteins).^22, 23^ TQ is an active component of *Nigella sativa* and acts as a multiple target modulator in cancer control via p53,^24^ nuclear factor-kappaB,^25^ protein kinase B suppression,^26^ caspase activation,^27^ and activation of tumour suppressor factor as well as peroxisome proliferator-activated receptor.^28^ In platelets, TQ induces apoptosis by increase of cytosolic calcium concentration, phosphoinositide 3-kinase and caspase-3 activation, ceramide formation, and mitochondrial depolarization.^29^

The mechanisms of Thr/Cvx-induced platelet activation and pro-coagulant activity are well characterised^13, 30, 31, 32^ and we used this model as a positive control to compare PKA/PKG effects on platelet apoptosis induced by other stimuli.

Here, we show that activation of PKA/PKG did not prevent ABT-737- and TQ-induced platelet apoptosis. In contrast, both ABT-737 and TQ activated PKA by cAMP-independent but caspase-3-dependent mechanisms and strongly inhibited thrombin-induced platelet activation.

## Results

### ABT-737and TQ induce platelet apoptosis, whereas Thr/Cvx induces pro-coagulant platelets

**Figure 1:**
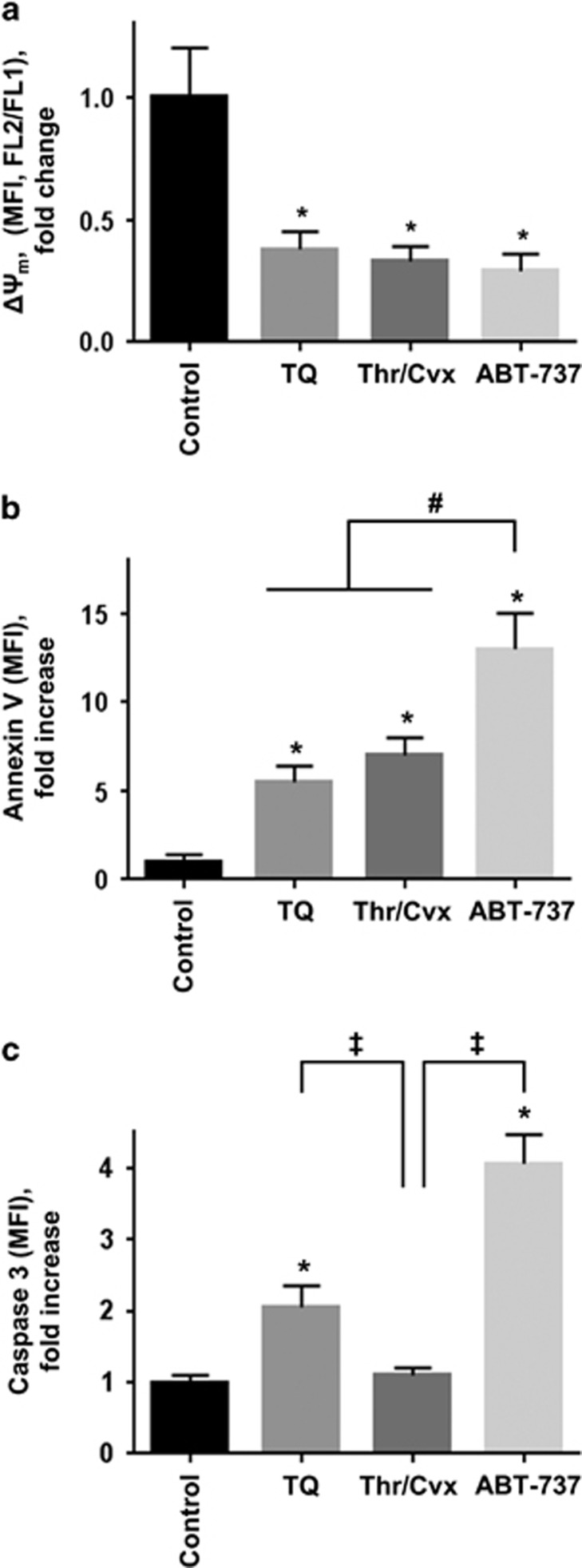
Platelet apoptotic events induced by ABT-737, TQ, and Thr/Cvx. Washed human platelets (1 × 10^7^/ml) were incubated with ABT-737 (1 *μ*M, 10 min), TQ (40 *μ*M, 60 min), or a combination of thrombin (Thr, 0.005 U/ml) and convulxin (Cvx, 5 ng/ml) for 2 min. (**a**–**c**) FACS analyses of mitochondrial membrane potential (ΔΨ_m_) (**a**), PS exposure (Annexin V binding (**b**), and caspase-3 activation (**c)** are shown. Data are presented as fold changes of mean fluorescence intensity (MFI) compared to control (considered as 1) and as mean±S.E.M., *n*=5; *P<0.05 compared to control, ^#^*P*<0.05 compared to ABT-737, ^‡^*P*<0.05 compared to Thr/Cvx

First, optimal incubation times and concentrations of the compounds required to induce apoptotic or pro-coagulant platelets were established (data not shown). In all experiments, platelets were treated with ABT-737 (1 *μ*M) or TQ (40 *μ*M) for 10 and 60 min or with a combination of Thr (0.005 U/ml) and Cvx (5 ng/ml) for 2 min. All compounds decreased ΔΨ_m_ to approximately 30% compared to untreated platelets (Figure 1a). ABT-737 induced strongest PS exposure (13±4-fold increase compared to control), whereas TQ (5.5±0.9) and Thr/Cvx (7.1±1.0) induced PS exposure was significantly less pronounced (Figure 1b). Both TQ and ABT, but not Thr/Cvx evoked significant caspase-3 activation (Figure 1c). These experiments confirmed that under the tested conditions Thr/Cvx treatment caused caspase-3-independent formation of pro-coagulant platelets, whereas ABT-737 and TQ induced caspase-3-dependent platelet apoptosis.

### PKA/PKG activation inhibits Thr/Cvx-triggered pro-coagulant state, but not ABT-737- or TQ-induced platelet apoptosis

**Figure 2:**
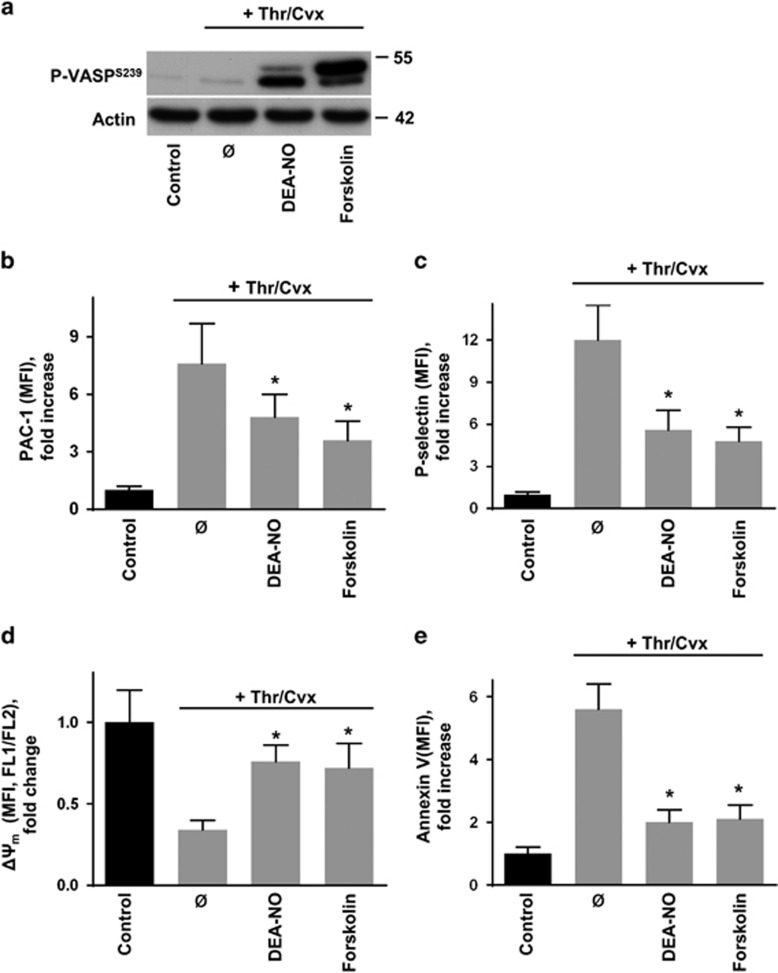
PKA and PKG activation inhibits Thr/Cvx-induced platelet activation and procoagulant activity. Washed human platelets (1 × 10^7^/ml) were treated with a combination of thrombin (Thr, 0.005 U/ml) and convulxin (Cvx, 5 ng/ml) for 2 min without (ø) or with DEA-NO (1 *μ*M, 2 min) or forskolin (5 *μ*M, 2 min) preincubation. (**a**) PKA/PKG activity was monitored using western blot analysis of VASP Ser239-phosphorylation (P-VASP^239^). Actin served as loading control. The presented western blot is representative of five independent experiments. (**b**–**e**) FACS analysis of integrin *α*IIb*β*3 activation (PAC-1 binding (**b**), P-selectin exposure **(c)**, ΔΨ_m_ (**d**), and PS-exposure (Annexin V binding (**e**). Data in (**b**–**e**) are fold increase of mean fluorescence intensity (MFI) compared to control (considered as 1) and are presented as mean±S.E.M., *n*=5; *P<0.05 compared to Thr/Cvx-stimulated platelets without DEA-NO or forskolin preincubation (ø)

Next, we investigated the impact of PKA or PKG activation on Thr/Cvx-triggered pro-coagulant state and on ABT-737- or TQ-induced platelet apoptosis. Kinase activities were monitored using the well-established and highly sensitive detection of VASP S239-phosphorylation (P-VASP^S239^), which has been used in numerous studies.^16, 33, 34^ In platelets, both PKA and PKG equally phosphorylate VASP at Ser239. Thr/Cvx stimulation alone did not induce VASP phosphorylation. However, activation of PKA by forskolin or PKG by the nitric oxide (NO) donor DEA-NO triggered strong VASP phosphorylation (Figure 2a) and significantly reduced the Thr/Cvx-induced platelet activation as revealed by a decreased integrin *α*IIb*β*3 activation (PAC-1 binding; Figure 2b) and diminished surface expression of P-selectin (Figure 2c). In the same experiments, PKA/PKG activation significantly attenuated the Thr/Cvx-induced platelet pro-coagulant state as revealed by a prevented decrease of ΔΨ_m_ (Figure 2d) and a blocked PS exposure (Figure 2e).

**Figure 3:**
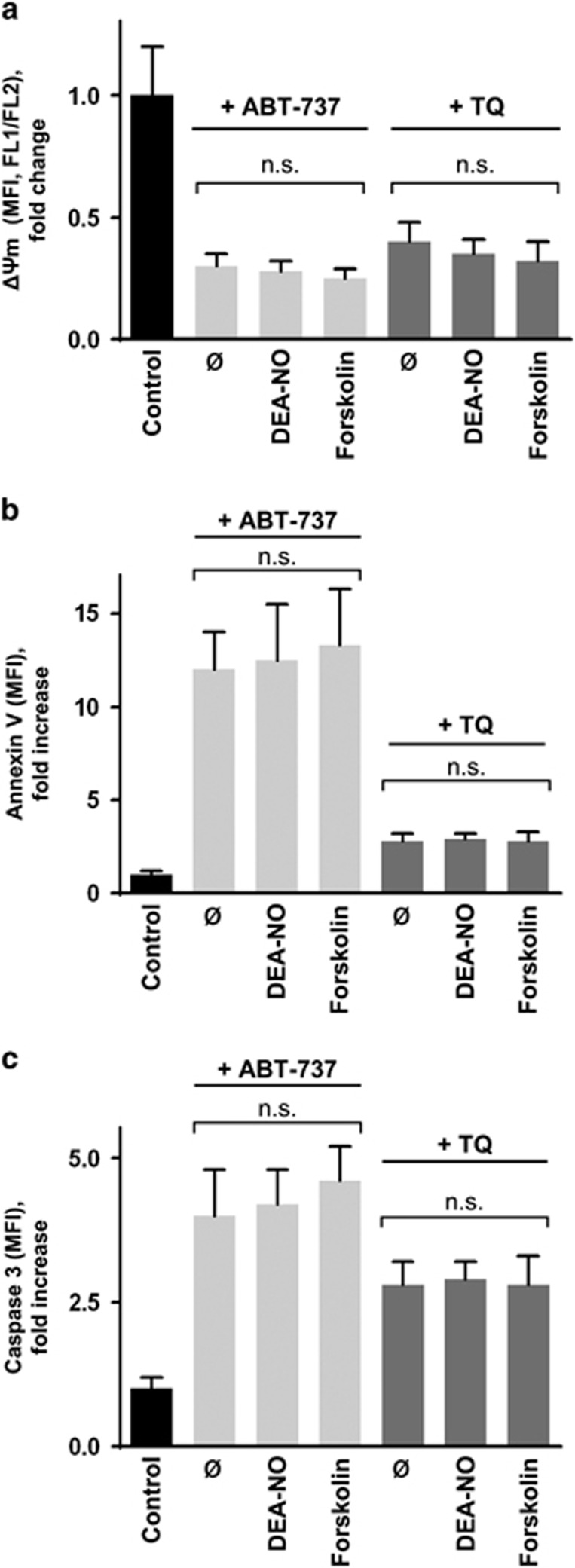
PKA or PKG activation does not prevent ABT-737- and TQ-induced platelet apoptosis. Washed human platelets (1 × 10^7^/ml) were treated with ABT-737 (1 *μ*M, 60 min) or TQ (40 *μ*M, 10 min) without (ø) or with DEA-NO (1 *μ*M, 2 min) or forskolin (5 *μ*M, 2 min) preincubation. (**a**–**c**) FACS analyses of ΔΨ_m_ (**a**), PS exposure (Annexin V binding (**b**), and caspase-3 activation (**c**) are shown. Data in (**a**–**c**) are fold changes of mean fluorescence intensity (MFI) compared to control (considered as 1) and are presented as mean±S.E.M., *n*=5, n.s. no significant difference compared to stimulated platelets without DEA-NO or forskolin preincubation (ø)

In a similar experimental set-up, we tested the effects of PKA/PKG activation on caspase-mediated apoptosis induced by ABT-737 and TQ. In contrast to Thr/Cvx-treatment, PKA/PKG activation did not reverse changes in ΔΨ_m_ (Figure 3a), PS exposure (Figure 3b), and caspase-3 activation (Figure 3c).

### ABT-737 and TQ induce VASP phosphorylation in platelets

**Figure 4:**
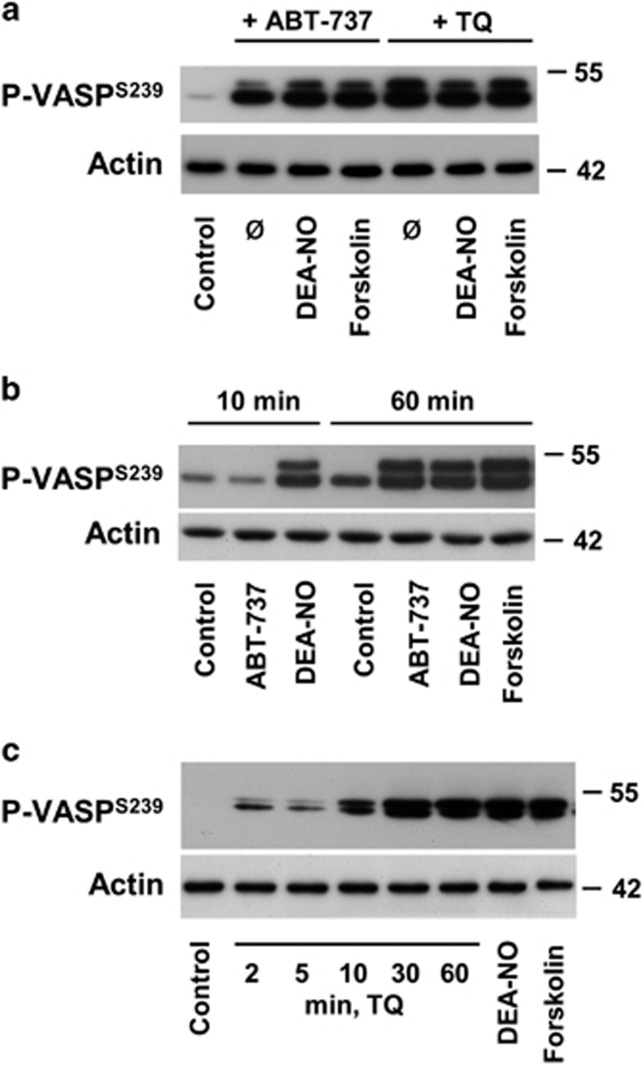
ABT-737 and TQ induce VASP phosphorylation in platelets. (**a**) Washed human platelets (3 × 10^8^/ml) were stimulated with ABT-737 (1 *μ*M, 60 min) or TQ (40 *μ*M, 10 min), or in combination with DEA-NO (1 *μ*M) or forskolin (5 *μ*M) for the same time. (**b**) Platelets were stimulated with the same concentrations (as in **a**) of ABT-737 and DEA-NO, or forskolin for indicated time. (**c**) Platelets were stimulated by TQ for indicated time or with DEA-NO (1 *μ*M), or forskolin (5 *μ*M) for 2 min. PKA/PKG activity was monitored using western blot analysis of VASP Ser239-phosphorylation (P-VASP^239^). Actin served as loading control. Presented western blot results are representative of five independent experiments

Surprisingly, we found that stimulation of platelets with ABT-737 or TQ induced strong VASP phosphorylation. The magnitude of VASP phosphorylation was comparable to the activation of PKG by DEA-NO or PKA by forskolin stimulation (Figures 4a–c), indicating a possible activation of one or both of the kinases by these compounds. Indeed, co-incubation with forskolin or DEA-NO did not further increase VASP phosphorylation levels (Figure 4a). We also investigated the kinetics of TQ/ABT-737-induced VASP phosphorylation (Figures 4b and c). TQ- and ABT-737-induced VASP phosphorylation was slow compared to the DEA-NO- and forskolin-induced VASP phosphorylation, which was already maximal after 2 min at the latest (compare Figure 2a). ABT-737-induced VASP phosphorylation was still low at 10 min and peaked at 60 min of incubation. TQ-dependent VASP phosphorylation reached half-maximal levels after 10 min and a sustained and maximal phosphorylation was observed at 30 and 60 min. DEA-NO stimulation of the soluble guanylate cyclise (GC) increases cGMP content, while direct activation of the adenylate cyclase (AC) by forskolin increases cAMP content. Both reactions are very fast and reached the maximum during 1–2 min. The slower kinetics of TQ/ABT-737-induced VASP phosphorylation may therefore argue for an alternative mechanism of kinase activation.

### ABT-737- and TQ-induced inhibition of platelet activation correlates with VASP phosphorylation

**Figure 5:**
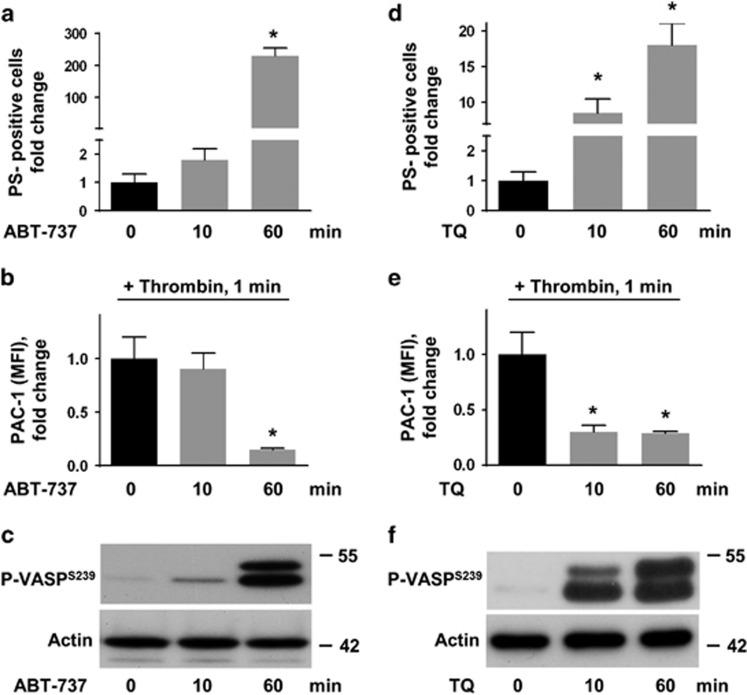
Inhibition of thrombin-induced platelet activation by ABT-737 and TQ correlates with VASP phosphorylation. Washed human platelets (1 × 10^8^/ml) were treated with ABT-737 ((**a**–**c**), 1 *μ*M) or TQ ((**d**–**f**), 40 *μ*M) for 0, 10, and 60 min as indicated in the corresponding western blots (**c** and **f**). In (**b** and **e**), platelets were pre-stimulated with thrombin (0.01 U/ml, 1 min). FACS analyses of the number of PS-positive platelets (Annexin V binding; (**a**,**d**) and integrin *α*IIb*β*3 activation PAC-1 binding; (**b**,**e**)) are shown. Data are fold increase of mean fluorescence intensity (MFI) compared to platelets without ABT-737 or TQ stimulation (0 min) and presented as mean±S.E.M., *n*=5; *P<0.05 compared to cells without stimulation. PKA/PKG activation was identified in these samples using western blot analysis of VASP phosphorylation. Actin served as loading control (**c**,**f**). Presented western blot results are representative of five independent experiments

ABT-737 was shown to prevent platelet activation despite PS exposure and thrombin generation.^6^ First, we confirmed that ABT-737 and TQ themselves did not induce platelet activation (integrin activation assessed by PAC-1 binding and P-selectin exposure) during 10 and 60 min incubation (data not shown). ABT-737 induced PS exposure (Annexin V binding) and abolished thrombin-triggered integrin activation (PAC-1 binding) after 60 min of treatment, but did not affect the activation at 10 min (Figures 5a and b). Therefore, the inhibition of platelet activation and increase of PS-exposure directly correlated with the time course of VASP phosphorylation, which was still low after 10 min but maximal after 60 min of ABT-737 incubation (Figure 5c). In case of TQ-treatment, PS-exposure and inhibition of integrin activation were already significantly changed after 10 min and this effect sustained during 60 min of incubation (Figures 5d and e). Again, inhibition of platelet activation and increase of PS-exposure directly correlated with TQ-induced VASP phosphorylation, which followed the same kinetics (Figure 5f).

**Figure 6:**
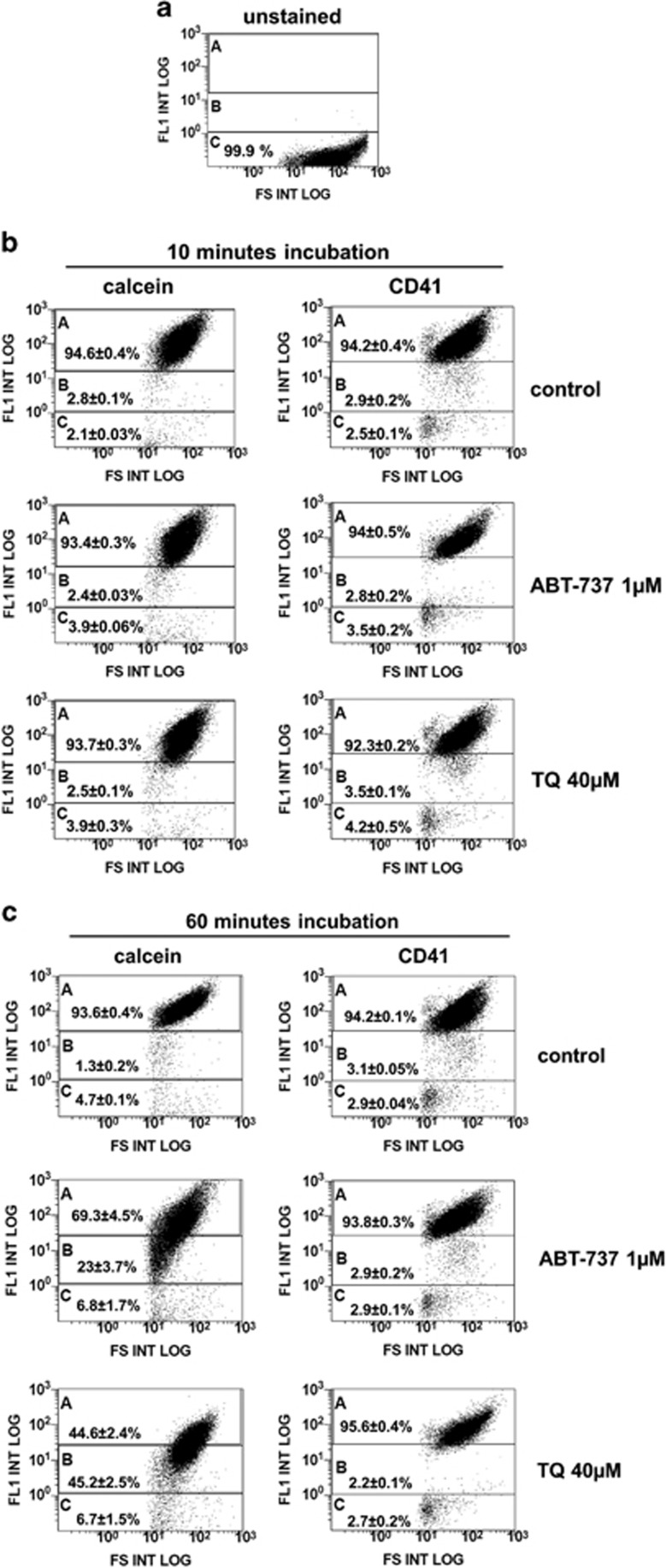
Impact of ABT-737- and TQ-treatment on platelet viability and integrin *α*IIb*β*3 surface expression. Washed human platelets (1 × 10^8^/ml) were treated with ABT-737 (1 *μ*M) or TQ (40 *μ*M) for the indicated times and analysed by FACS for viability (calcein-fluorescence) and integrin *α*IIb*β*3 surface expression (CD41). All platelets were separated into three gates: gate A represents more than 93% calcein- and CD41-positive platelets in control samples, gate C corresponds to unstained platelets (calcein-negative/dead cells; or CD-41-negative platelets; compare (**a**)) and gate B represents platelets with intermediate calcein- or CD41 fluorescence. Distribution of platelets in each gate was calculated as a percent of all analysed (50 000) events taken as 100%. Data are presented as mean±S.E.M., *n*=4. (**a**–**c**) represent a set of original FACS data representative of four independent experiments

Besides kinase activation, the observed inhibition of platelet activation by ABT-737 or TQ may also be related to reduced platelet viability and/or integrin surface expression. We used the vital dye calcein-AM, a fluorogenic substrate of intracellular esterases, for the reliable assessment of platelet viability^35, 36^ and *α*IIb*β*3 integrin/CD41-specific antibodies to determine the surface expression of the glycoprotein by FACS. After 10 min of incubation with ABT-737 or TQ, platelet viability and CD41 surface expression were statistically undistinguishable from control values (Figures 6a and b). This is especially noteworthy for the TQ-treatment, because inhibition of platelet activation by TQ was nearly maximal under this condition (Figure 5e). After 60 min of ABT-737 or TQ treatment, reduced esterase activity was observed in 23% and 45% of the platelets, respectively. However, the percentage of calcein-negative/dead cells was still low, 6.8% and 6.7%, respectively, and did not significantly differ from the control group (4.7% Figure 6c). Importantly, CD41 surface expression was again completely unaffected by ABT-737 or TQ incubation. Taken together, the data indicate that ABT-737 or TQ-mediated platelet inhibition of thrombin-induced activation (more than 70%, Figures 5b and e) is not directly related to a reduced platelet viability or integrin *α*IIb*β*3 surface expression.

### ABT-737 and TQ do not increase cAMP or cGMP levels in human platelets

**Figure 7:**
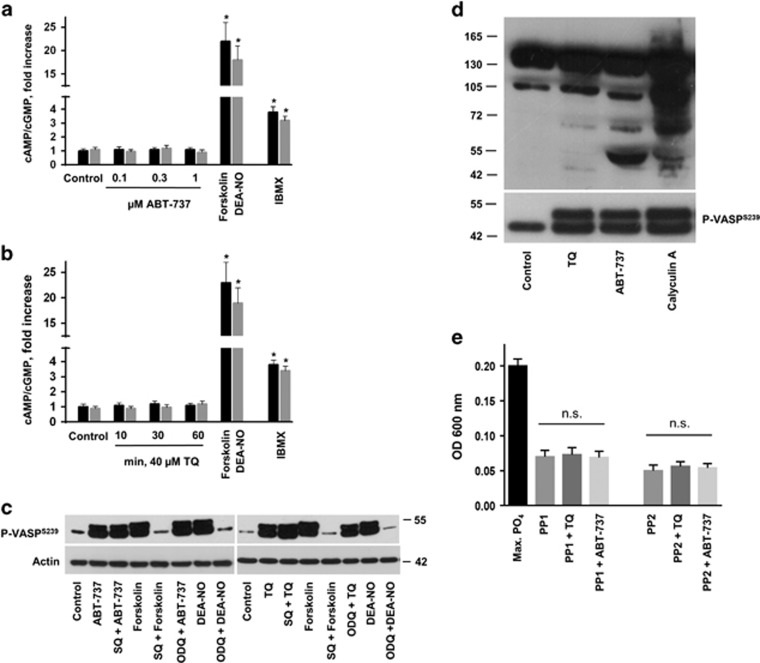
No impact of ABT-737 and TQ on cyclic nucleotide levels or phosphatase activity. (**a**,**b**) Washed human platelets (3 × 10^8^/ml) were incubated with indicated concentrations of ABT-737 for 60 min (**a**), or for the indicated time with 40 *μ*M TQ (**b**) and then prepared for cGMP/cAMP quantification by competitive immunoassay kit. Cells treated with forskolin (5 *μ*M), DEA-NO (1 *μ*M) for 1 min, or IBMX (50 *μ*M) for 10 min were used as positive controls. Data are presented as fold increase compared to control set to 1 (mean±S.E.M., *n*=5, *P<0.05 compared to control). (**c**) Washed human platelets were stimulated with ABT-737 (1 *μ*M, 60 min), TQ (40 *μ*M, 10 min), forskolin (5 *μ*M, 1 min), DEA-NO (0.1 *μ*M, 1 min) with or without preincubation with inhibitors of AC (SQ22536, 100 *μ*M, 10 min) or GC (ODQ, 10 *μ*M, 10 min). Samples were analysed by western blot for VASP^Ser239^ phosphorylation. Actin blots served as loading control. Presented western blot results are representative of five independent experiments. (**d**) Washed human platelets (3 × 10^8^/ml) were stimulated with ABT-737 (1 *μ*M), TQ (40 *μ*M), or Calyculin A (1 *μ*M) for 60 min. Platelet lysates were then analysed by western blot with phospho-Ser/Thr-specific antibodies (upper panel) or P-VASP^239^-specific antibodies (lower panel). Data are representative of at least four independent experiments. (**e**) *In vitro* phosphatase assay: the PP1A/PP2A-driven release of free phosphate from a defined phosphopeptide after 15 min incubation (corresponding to 50% phosphate release of maximal phosphate release measured after 1 h incubation with PP1A or PP2A) was measured in the presence and absence (ø) of TQ or ABT-737, using a colorimetric reaction at 600 nm as described in the method part. Data are presented as optical density (OD) at 600 nm (mean±S.E.M., *n*=3; n.s. no significant difference compared to phosphatase reactions without TQ/ABT-737 supplementation)

PKA and PKG are activated by elevated cAMP and cGMP levels, respectively. Therefore, we measured whether ABT-737 and TQ elevate cyclic nucleotides by activating cyclases or by inhibiting cyclic nucleotide hydrolysing phosphodiesterase (PDE) in platelets. DEA-NO and forskolin directly stimulate the GC and AC, respectively, and hence were used as positive controls while the non-specific PDE inhibitor IBMX was used as a negative control. Unexpectedly, under conditions which strongly induced ABT-737 and TQ-dependent VASP phosphorylation (Figure 5), we did not observe any increase in cyclic nucleotide concentrations (Figures 7a and b). To confirm that ABT-737 and TQ did not activate AC or GC we used AC inhibitor SQ22536 and GC inhibitor ODQ. Pre-incubation of platelets with SQ22536 and ODQ completely blocked forskolin and DEA-NO-induced VASP phosphorylation, but had no effect on ABT-737 or TQ-induced VASP phosphorylation (Figure 7c).

### ABT-737 and TQ do not activate PP1 and PP2 phosphatases

VASP phosphorylation is dynamically regulated by the kinase activity of PKA/PKG on the one hand and the phosphatase activity of protein phosphatase 1A (PP1A) and protein phosphatase 2A (PP2A) on the other hand.^37^ Because increased VASP phosphorylation by ABT-737 and TQ could be an indirect consequence of Ser/Thr phosphatase inhibition, we tested the impact of the compounds on Ser/Thr phosphatase activity. For this, we used two different approaches. In the first experiment, platelets were incubated with ABT-737, TQ, and Calyculin A (CA), a well-established Ser/Thr phosphatase inhibitor, and platelet lysates were analysed by Western blot with phospho-Ser/Thr specific antibodies. While all three compounds induced strong VASP phosphorylation, only incubation with CA resulted in numerous additional phospho Ser/Thr proteins of different molecular weight (Figure 7d). After ABT-737 or TQ treatment, however, only few Ser/Thr phosphorylated proteins emerged on the blot, indicating no strong effect of both compounds on phosphatases activity in intact cells. To confirm this with an independent approach, we directly tested whether ABT-737 or TQ could inhibit the phosphatase activity of PP1A and PP2A *in vitro*. In this assay, phosphatase activity was measured by free phosphate concentration released from a phosphatase-specific substrate. However, neither ABT-737 nor TQ did affect Ser/Thr phosphatase activity of PP1A or PP2A (Figure 7e).

### ABT-737 and TQ activate PKA by a caspase-3 dependent mechanism

**Figure 8:**
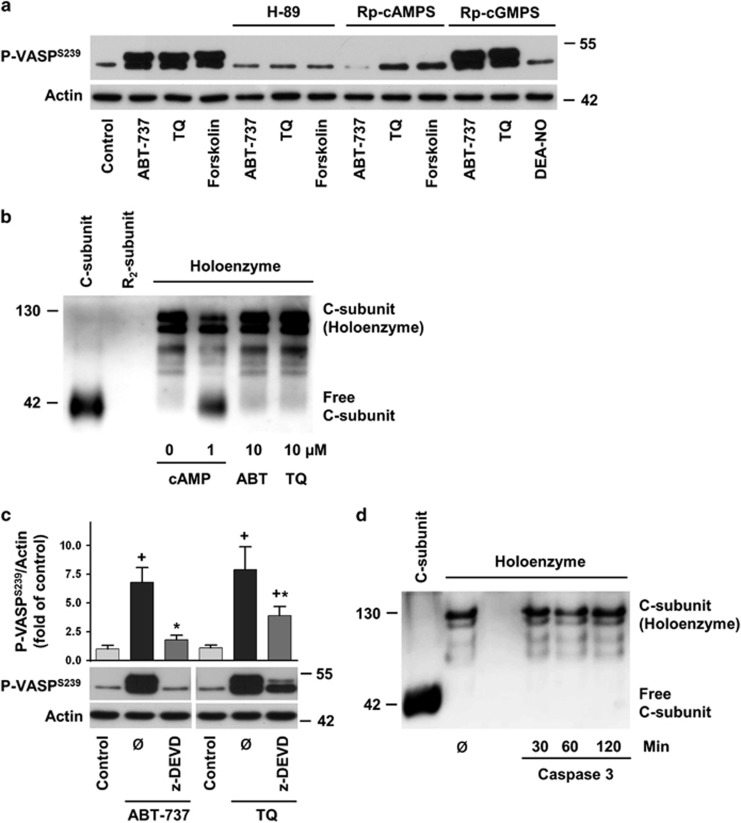
ABT-737 and TQ activated PKA in platelets by a caspase-3-dependent mechanism. (**a**) Washed human platelets were stimulated with ABT-737 (1 *μ*M, 60 min), TQ (40 *μ*M, 10 min), forskolin (5 *μ*M, 1 min), or DEA-NO (0.1 *μ*M, 1 min) with or without preincubation with PKA inhibitors H-89 (10 *μ*M, 5 min) or Rp-8-Br-cAMPS (Rp-cAMPS, 200 *μ*M, 10 min), or PKG inhibitor Rp-8-Br-PET-cGMPS (Rp-cGMPS, 200 *μ*M, 10 min). Samples were analysed by Western blot for VASP^Ser239^ phosphorylation. Presented data are representative of four independent experiments. (**b**) PKA II holoenzyme was incubated in the absence (control) or presence of 1 *μ*M cAMP, 10 *μ*M ABT-737 or 10 *μ*M TQ for 30 min. Proteins were resolved on a 10% native acrylamide gels and processed for western blotting with PKAC antibodies. (**c**) Washed human platelets were stimulated as indicated without (ø) or with preincubation with caspase-3 inhibitor (zDEVDfmk, 100 *μ*M, 5 min). Samples were analysed by western blot for VASP^Ser239^ phosphorylation. For the bar graphs, immunoblots of four independent experiments were scanned and quantified with ImageJ. The intensity of the VASP^Ser239^ signal was normalized to the actin signal, which was designated as 1 in control samples. Results are means±S.E.M., *n*=4, ^+^significant differences (*P*<0.05) compared to control samples; * significant differences (*P*<0.05) compared to ABT-737 or TQ samples without caspase-3 inhibitor. (**d**) PKA II holoenzyme was incubated with active caspase-3 for the indicated times. Proteins were resolved on a 10% native acrylamide gels and processed for Western blotting with PKAC antibodies. Presented data are representative of three independent experiments

So far, our data demonstrate that ABT-737 and TQ induce VASP phosphorylation independent of cyclic nucleotides and also independent of phosphatase activity. To distinguish between PKA and PKG activation, we studied ABT-737/TQ-induced VASP phosphorylation in platelets after pre-incubation with membrane permeable PKA- (H89 and Rp-8-Br-cAMPS) and PKG-inhibitors (Rp-8-Br-PET-cGMPS).^38^ Pre-incubation of platelets with H89 and Rp-8-Br-cAMPS strongly inhibited ABT-737-, TQ-, and forskolin-induced VASP phosphorylation, while Rp-8-Br-PET-cGMPS only inhibited DEA-NO-induced VASP phosphorylation (Figure 8a). The data clearly indicate that VASP phosphorylation is mediated by PKA, but not PKG activation. PKA is comprised of two catalytic (C) subunits that bind to a regulatory subunit dimer to form the inactive holoenzyme. For PKA activation, the C-subunits are released from the regulatory subunits. To test whether ABT-737 or TQ can directly activate PKA we incubated both compounds with PKA holoenzyme *in vitro* and studied release of the catalytic subunits. Incubation of PKA with cAMP resulted in strong release of the free C-subunit. In contrast, neither ABT-737 nor TQ increased free C-subunit levels as observed in native gels (Figure 8b), indicating that both compounds are not direct PKA activators *in vitro*. Because ABT-737 and TQ activated caspases, we tested whether PKA activation might be caspase-dependent. Pre-incubation of platelets with pan-caspase (z-VAD-fmk, data not shown) and caspase-3 specific (z-DEVD-fmk) inhibitors completely blocked ABT-737-induced VASP phosphorylation, whereas TQ-induced phosphorylation was only partly inhibited (Figure 8c). We also tested whether active caspase-3 can activate PKA *in vitro* and incubated PKA holoenzyme with active caspase-3. However, active caspase-3 did not release C-subunit from the holoenzyme (Figure 8d and supplementary Figure 1), indicating that caspase-3 cannot directly activate PKA *in vitro*.

Together, these data suggested that ABT-737- and TQ-induced VASP phosphorylation in intact platelets could be, at least partly, mediated by cAMP-independent and caspase-3-dependent mechanisms of PKA activation.

## Discussion

Thrombocytopenia and bleeding disorders are the primary, dose-limiting adverse effects of anticancer therapy. Recent data from mouse and human platelets revealed that activation of PKA/PKG not only inhibited Thr/Cvx-induced platelet activation but also its pro-coagulant state.^13^ In the present study, we tested whether or not PKA/PKG activation could inhibit caspase-dependent apoptosis induced by ABT-737 (navitoclax) or thymoquinone (TQ), thereby limiting chemotherapy-induced thrombocytopenia. This is especially important given that activation of cyclic nucleotide signalling in combination chemotherapy is an emerging strategy in cancer treatment.^39, 40, 41^ However, PKA/PKG-activation did not block caspase-3-dependent platelet apoptosis induced by the compounds (Figures 3b and c). In contrast, both substances induced PKA activation themselves and PKA activation (Figure 4) correlated with platelet inhibition and apoptosis (Figure 5). We tested whether ABT-737- and TQ-induced PKA activation is mainly responsible for the inhibition of thrombin-induced platelet activation, or if this effect may be a consequence of the decreased platelet viability or integrin *α*IIb*β*3 surface expression. However, neither treatment with TQ or ABT-737 significantly changed the number of dead (calcein-negative) platelets or integrin *α*IIb*β*3 surface expression in platelets compared to control cells (Figure 6) under conditions that showed maximal platelet inhibition and PKA activation (VASP phosphorylation, Figures 5e and f).

The PKA holoenzyme in its inactive form is a tetramer consisting of two regulatory subunits (R) and two catalytic subunits (C). Both R subunits contain two binding sites for cAMP. Upon binding of cAMP the two C subunits are released and activated to phosphorylate their substrates. In addition to this ‘classical’ cAMP-dependent PKA activation, at least three other cAMP-independent mechanisms are described: binding of PKA-C-subunit to IkB in NFkB-IkB complex,^42, 43^ interaction of p90 ribosomal S6 kinase (RSK1) with PKARI*α*,^44^ and transforming growth factor beta-induced Smad3/Smad4 complex formation.^45^ Furthermore, some small molecules can cAMP-independently activate PKA.^46, 47^ However, molecular mechanisms, how these substances directly activate PKA are not known.

In 1987, VASP was discovered and characterized as the major PKA/PKG substrate in platelets.^33, 48^ Three decades later, measurement of VASP phosphorylation status has become a highly sensitive and reliable assay, which is used in numerous studies to assess PKA/PKG-dependent effects in cardiovascular cells and which is widely used in clinical diagnosis of platelet reactivity.^16, 49, 50, 51, 52, 53^ In our study, we used VASP phosphorylation as a marker of ABT-737- or TQ-induced PKA activation in platelets. Interestingly, VASP phosphorylation correlated well with platelet inhibition and apoptosis. Whether or not VASP phosphorylation plays a functional role in caspase-dependent platelet apoptosis, remains unknown. In apoptotic platelets, we did not observe apparent VASP cleavage, indicating that VASP itself is not a prominent substrate for caspase-3. However, there is at least circumstantial evidence for a role of VASP in caspase-3-mediated membrane blebbing, a hallmark of apoptotic events. Cleavage of the plasma membrane-associated spectrins by caspase-3 leads to membrane blebbing, the formation of apoptotic bodies, and irreversible cell death. In endothelial cells, we have previously shown that VASP binding to *α*II-spectrin attenuates *α*II-spectrin cleavage in apoptotic cells and that PKA-induced VASP phosphorylation regulates this process.^54, 55^ Whether a similar mechanism exists in platelets is unclear. However, given that spectrin disruption destabilizes pro-platelets, causing blebbing and swelling,^56^ it is tempting to speculate that VASP phosphorylation may be of functional importance for platelet apoptosis.

In our platelet experiments, ABT-737- and TQ-induced PKA activation was, at least partially, blocked by caspase-3 inhibitors (Figure 8c). PKA has been implicated in regulating caspase activity,^57, 58^ but there have been no reports of a reverse role for caspases in PKA activation. In nucleated cells, caspase-3 activation was shown to increase phosphatase PP2A activity by cleavage of the regulatory subunit.^59^ We tested whether or not a similar mechanism may contribute to PKA activation. In our *in vitro* experiments, however, active caspase-3 in combination with ABT-737 or TQ failed to activate PKA, indicating that additional intracellular signalling pathways are required for PKA activation after treatment with the compounds.

A role of cAMP signalling in regulation of apoptosis is known for decades. However, cAMP can either stimulate or inhibit apoptosis in a cell type-dependent manner and the underlying mechanisms remain elusive. In nucleated cells, exchange protein activated by cAMP (Epac) is a second effector of cAMP-signalling that seems to mediate anti-apoptotic effects.^20^ However, these cAMP effects are unlikely to play a role in platelets because Epac is not expressed in platelets^60^ and there was no increase of cAMP levels in ABT-737- or TQ-treated platelets. On the other hand, substantial evidence indicates that pro-apoptotic events of cAMP in nucleated cells are, at least in part, mediated by the PKA-dependent phosphorylation of protein targets.^61, 62^ Given that the effects of ABT-737, TQ, and PKA on nucleated cell apoptosis partially involve regulation of gene expression, it is difficult to compare these findings to the situation in platelets. For example, in Jurkat T-cells, TQ downregulated the expression of phosphodiesterases PDE1, PDE3, and PDE4 during 24 h of incubation, which increased cGMP levels and reduced cAMP concentrations.^63^ In platelets, we did not see any effect of TQ or ABT-737 on cyclic nucleotide levels. All these data indicate that regulation of cyclic nucleotides as well as PKA/PKG activity by ABT-737 and TQ is cell type dependent and involves different molecular mechanisms. Whether long-term PKA/PKG activation can influence platelet apoptosis and how ABT-737 or TQ cAMP-independently activate PKA is not clear and should be addressed in a separate study.

In summary, we showed that elevation of cyclic nucleotides does not inhibit apoptosis induced by ABT-737 and TQ. In contrast, both anti-cancer drugs cAMP-independently activated PKA themselves, thereby inhibiting platelet activation. Therefore, our study provides novel insights underlying the high bleeding risk in chemotherapy patients.

## Materials and methods

### Materials

Thrombin (Roche, Mannheim, Germany), convulxin (Axxora, Lörrach, Germany), Forskolin, Calyculin A, H89, thymoquinone, ODQ, IBMX, and SQ22536 (Sigma-Aldrich, Munich, Germany), DEA-NO (Alexis Biochemicals, Lörrach, Germany), Rp-8-Br-cAMPS and Rp-8-Br-PET-cGMPS (BioLog, Bremen, Germany), phospho-VASP^S239^ (Nano Tools, Teningen, Germany), phospho Ser/Thr and actin antibodies (Cell Signaling, Frankfurt, Germany), horseradish peroxidase-conjugated anti-rabbit or anti-mouse IgG (Amersham, Freiburg, Germany), Annexin-V-PE, P-selectin, rabbit anti-active caspase-3-PE, CD41-FITC, and PAC-1-FITC antibodies (BD-Bioscience, Heidelberg, Germany), JC1 (Invitrogen, Eugen, Germany), ABT-737 (Selleckchem, Munich, Germany), z-VAD-fmk and z-DEVD-fmk (Calbiochem, Schwalbach, Germany), calcein-AM (Molecular Probes, Gottingen, Germany) were purchased. PKA type II holoenzyme was purified from bovine heart as described.^64^ C-subunit of PKA antibody was a kind gift of G. Schwoch, Göttingen (Germany). Active caspase-3 (ENZO ALX-201-059) and caspase-3-specific substrate Ac-DEVD-pNA (ENZO ALX-260-033) were from ENZO Life Sciences (Lörrach, Germany).

### Human platelet preparation

Human platelets were prepared and used as previously reported.^43, 65^ Blood was obtained from healthy volunteers according to our institutional guidelines and the Declaration of Helsinki. Our studies with human platelets were approved and reconfirmed by the local ethics committee of the University clinic of Wuerzburg (Studies No. 67/92 and 114/04).

### FACS analysis

FACS analysis was performed using a Becton Dickinson FACS Calibur with CELLQuest software, version 3.1f. For the detection of surface PS, *α*IIb*β*3 integrins surface expression, or activated *α*IIb*β*3 integrins, washed platelets (50 *μ*l) were labelled with Annexin V-PE or PAC-1-FITC for 10 min at RT after stimulation. The platelets were then diluted with Annexin V-binding solution (140 mM NaCl, 10 mM HEPES, and 2.5 mM CaCl_2_) for Annexin V or PBS for PAC-1, or CD41 and immediately analysed by flow cytometry. Caspase-3 activity was measured by rabbit anti-active caspase-3-PE antibody according to the manufacturer’s instruction.

### Analysis of platelet viability by calcein-AM staining and *α*IIb*β*3 integrins surface expression

For FACS analysis of platelet viability, platelets were loaded with 0.1 *μ*M calcein-AM for 30 min, incubated without (control) or with ABT-737 or TQ at 37 °C for indicated time and then analysed for intracellular esterase activity that liberates green fluorescence calcein from the acetoxymethyl (AM) ester group leading to the accumulation of the dye within viable cells.^35^ Platelets were divided into three gates with different fluorescence intensity. Gate A contains calcein positive platelets, gate B contains platelets with reduced calcein fluorescence and gate C represents calcein-negative platelets corresponding to unstained cells. *α*IIb*β*3 integrins surface expression was analysed similarly by CD41-specific antibodies directed against the *α*IIb chain. Distribution of platelets in each gate was calculated as percent of all analysed (50 000) events taken as 100%.

### Analysis of mitochondrial membrane potential

Washed platelets (10^7^ platelets/ml) were stimulated and then incubated with JC1 dye for 10 min at RT. Mitochondrial membrane potential (ΔΨ_m_) was analysed by FACS. Green fluorescence (FL1) and red fluorescence (FL2) were measured in logarithmic scales using voltage settings of 710 or 588 respectively. Compensation settings were performed using FITC- and PE-labelled beads (BD-Bioscience). JC1 is sensitive to ΔΨ_m_ and the ratio of fluorescence in FL2 to FL1 corresponds to changes of ΔΨ_m_.

### Western blot analysis

For western blot analysis, washed platelets (3 × 10^8^ platelets/ml) were stimulated with indicated (time/concentration) compounds and then lysed with Laemmli sample buffer. Western blots were performed as described.^65^

### Determination of platelet cGMP and cAMP concentration

cGMP and cAMP content were determined in washed human platelets by a competitive immunoassay (Cayman Chemical, Hamburg, Germany) as per the manufacturer’s protocol.

### PP1A/PP2 *in vitro* phosphatase activity assay

Phosphatase (PP1A/PP2) assay was performed with a colorimetric assay following the manufacturer’s instructions (Biozol, Eching, Germany). Phosphatases were incubated with ABT-737 and TQ and the reaction was started by 200 *μ*M threonine phosphopeptide. After 15 min, reaction was stopped by Biomol Green (ENZO Life Sciences). After 30 min at RT, colour intensity was measured with absorbance at 600 nM in Wallac Victor (Perkin Elmer, Hamburg, Germany).

### PKA dissociation by ABT-737 and TQ (native gels)

PKA II holoenzyme (0.1 *μ*M, 150 ng) was incubated in phosphorylation buffer (10 mM HEPES pH 7.4, 5 mM MgCl_2_, 1 mM EDTA, 0.2 mM dithiothreitol) in the absence (control) or presence of 1 *μ*M cAMP, 10 *μ*M ABT-737 or 10 *μ*M TQ. Reaction was started by the addition of 100 *μ*M ATP and stopped after 30 min by the addition of 2 × sample buffer (62.5 mM Tris-HCl, pH 6.8, 25% glycerol, 1% bromophenol blue) without heating. Probes were loaded on a 10% acrylamide gel without SDS. Anode and cathode buffers were prepared and used as described.^66^ C-subunit of PKA was visualized by Western blotting.

### *In vitro* caspase-3 activity

Active caspase-3 (2U) with an activity of 2 nM substrate per hour at 37 °C was incubated with PKA II holoenzyme (0.1 *μ*M, 150 ng) in the buffer supplied by manufacturer. cAMP (1 *μ*M) was used as a positive control. Reaction was stopped after indicated times by the addition of 2 × sample buffer (62.5 mM Tris-HCl, pH 6.8, 25% glycerol, 1% bromophenol blue) without heating. Probes were loaded on a 10% acrylamide gel without SDS and processed for western blotting with PKA C-subunit antibody. Caspase-3 activity was measured colorimetrically at 405 nm by degradation of the specific substrate (Ac-DEVD-pNA) at indicated times.

### Data analysis

All experiments were performed at least four times, and the data were expressed as means±S.E.M. Differences between groups were analysed by ANOVA followed by Bonferroni’s test and Student *t*-test was used when appropriate. *P*<0.05 was considered statistically significant.
