# Supplementary material for: Protein kinase A activation by the anti-cancer drugs ABT-737 and thymoquinone is caspase-3-dependent and correlates with platelet inhibition and apoptosis
Source: Cell Death Dis. 2017 Jun 29;8(6):e2898–. doi: 10.1038/cddis.2017.290 (PMC5520940; doi:10.1038/cddis.2017.290)
Supplement: Supplementary Figure 1 [file cddis2017290x1.pdf]

Supplementary Figure

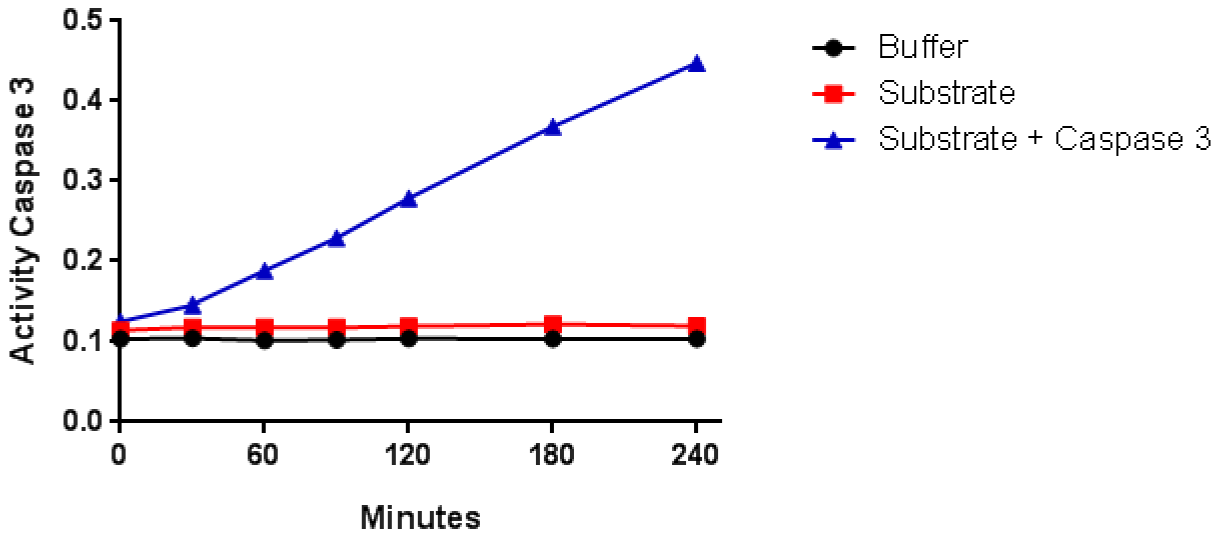

Supplementary Figure 1. Colorimetric analysis of caspase-3 activity

Activity of caspase 3 was measured colorimetrically at 405 nm by degradation of the specific substrate (Ac-DEVD-pNA) on indicated times. Presented data are representative of 3 independent experiments.
